# Early‐onset colorectal cancer in young individuals

**DOI:** 10.1002/1878-0261.12417

**Published:** 2018-12-22

**Authors:** Gianluca Mauri, Andrea Sartore‐Bianchi, Antonio‐Giampiero Russo, Silvia Marsoni, Alberto Bardelli, Salvatore Siena

**Affiliations:** ^1^ Niguarda Cancer Center Grande Ospedale Metropolitano Niguarda Dipartimento di Oncologia e Emato‐Oncologia Università degli Studi di Milano (La Statale) Milan Italy; ^2^ Epidemiology Unit Agenzia Tutela Salute della Città Metropolitana di Milano Milan Italy; ^3^ FIRC Institute of Molecular Oncology (IFOM) Milan Italy; ^4^ Department of Oncology University of Turin Italy; ^5^ Candiolo Cancer Institute – FPO IRCCS Turin Italy

**Keywords:** colorectal cancer, familial colorectal cancer, hereditary colorectal cancer, sporadic early‐onset colorectal cancer, young adults

## Abstract

Treatment of young adults with colorectal cancer (CRC) represents an unmet clinical need, especially as diagnosis in this population might lead to the greatest loss of years of life. Since 1994, CRC incidence in individuals younger than 50 years has been increasing by 2% per year. The surge in CRC incidence in young adults is particularly alarming as the overall CRC frequency has been decreasing. Early‐onset CRC are characterized by a more advanced stage at diagnosis, poorer cell differentiation, higher prevalence of signet ring cell histology, and left colon‐sided location of the primary tumor. Among EO‐CRC, approximately 30% of patients are affected by tumors harboring mutations causing hereditary cancer predisposing syndromes, and 20% have familial CRC. Most notably, the remaining 50% of EO‐CRC patients have neither hereditary syndromes nor familial CRC, thus representing a formidable challenge for research. In this review article we summarize epidemiology, clinical and molecular features, heredity and outcome of treatments of EO‐CRC, and provide considerations for future perspectives.

**Abbreviations**
AYA: adolescent and young adult
CIMP: CpG island methylator phenotype
CIN: chromosomal instability
CRC: colorectal cancer
EO‐CRC: early‐onset colorectal cancer
FAP: familial adenomatous polyposis
FOBT: fecal occult blood test
LS: Lynch syndrome
MACS: microsatellite and chromosome stable
MAP: MutYH‐associated polyposis
mCRC: metatastic colorectal cancer
MMR: mismatch repair
MSI: microsatellite instability
NAP: NTHL1‐associated polyposis
OS: overall survival
PAPP: polymerase proofreading‐associated polyposis
PFS: progression‐free survival
RR: Response rate
SEER: surveillance, epidemiology and end results program

## 1 Introduction

Colorectal cancer (CRC) is the third most common cancer and cause of cancer death worldwide in both genders (Siegel *et al*., 2018). At diagnosis, the median age of patients with colon cancer is 68 and 72 years in men and women, respectively; the median age of patients with rectal cancer is 63 years in both genders (American Cancer Society. Colorectal Cancer Facts & Figures, 2017; Siegel *et al*., 2018). In recent years, the overall CRC incidence and mortality in the USA and Europe has dropped (Malvezzi *et al*., 2018; Siegel *et al*., 2018). Since the mid‐2000s, CRC incidence in the USA has decreased by 2–3% per year in men and women (American Cancer Society. Colorectal Cancer Facts & Figures, 2017; Siegel *et al*., 2018). This reduction has been mainly correlated with the spread of screening tests [fecal occult blood test (FOBT) and colonoscopy] allowing detection and excision of premalignant lesions, and to a higher awareness of CRC risk factors among the population (Holme *et al*., 2013; Siegel *et al*., 2018; Welch and Robertson, 2016). Since 2012 in Europe, the CRC mortality rate decreased by 6.7% in men and 7.5% in women, whereas between 2008 and 2016, CRC incidence increased by 6% annually (Malvezzi *et al*., 2018; Vuik *et al*., 2018). Overall survival (OS) at 5 years from diagnosis is around 60% considering all stages of disease (Siegel *et al*., 2018; Van Cutsem *et al*., 2014). Metastatic CRC, despite therapeutic advances, exhibits poor prognosis; with the present state of the art, only 14% of patients are alive after 5 years from diagnosis (Siegel *et al*., 2018; Van Cutsem *et al*., 2014).

In 2010, CRC among patients < 50 years old accounted for 4.8% and 9.5% of colon and rectal cancers, respectively (Bailey *et al*., 2015; Silla *et al*., 2014). Interestingly, recent studies on several continents have reported an increase in CRC incidence in this age subset and especially in individuals < 40 years (Bailey *et al*., 2015; Bleyer *et al*., 2017; Exarchakou *et al*., 2018; Hessami Arani and Kerachian, 2017; Troeung *et al*., 2017), including also the metropolitan area of Milan, Italy (A.‐G. Russo, A. Andreano, A. Sartore‐Bianchi, G. Mauri, A. Decarli & S. Siena, personal communication). To date, the actual magnitude and the underlying etiologies of this increase are unclear. Diagnostic and therapeutic protocols dedicated to early‐onset CRC (EO‐CRC) in young individuals are a currently unmet clinical need. Also, there is no consensus on whether EO‐CRCs are undistinguishable or are a distinct molecular/immunologic entity from CRC in older patients (Bleyer *et al*., 2017; Luzzatto and Pandolfi, 2015; Tomasetti and Vogelstein, 2015; Tricoli *et al*., 2016).

In this article, we critically review clinical and molecular characteristics of EO‐CRC based on retrospective analyses, clinical trials, books and reviews currently available in the scientific literature. A systematic review approach has been applied for prognosis and standard metastatic colorectal cancer (mCRC) molecular biomarkers (*RAS* and *BRAF*) prevalence among EO‐CRC.

## 2 Materials and methods

**Figure 1:**
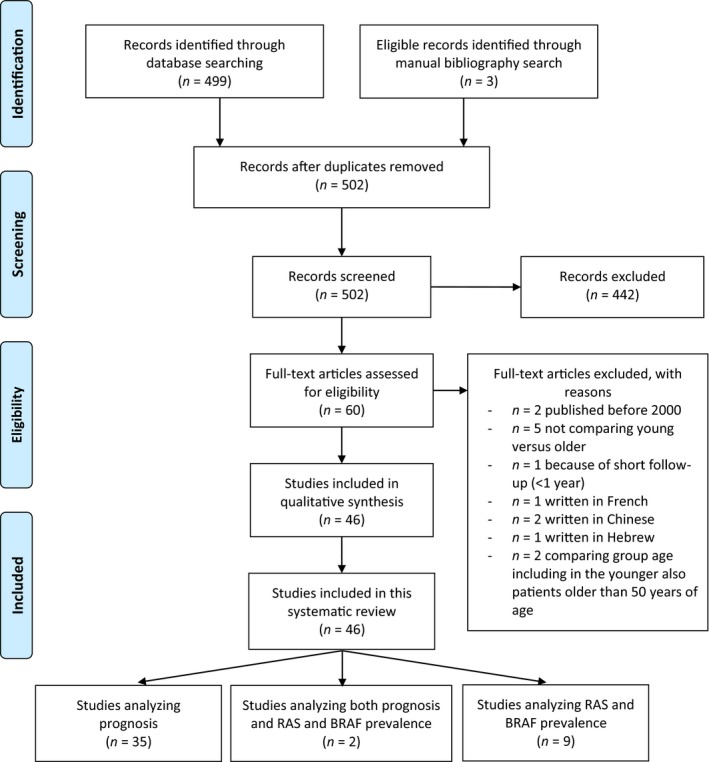
PRISMA 2009 flow diagram depicting the systematic review process performed to retrieve articles on prognosis and RAS and BRAF prevalence among EO‐CRC.

We first performed a narrative review of clinical and pathological characteristics of EO‐CRC, retrieving articles through PubMed database, American Society of Clinical Oncology, European Society for Medical Oncology, and United European Gastroenterology meeting libraries, websites www.cancer.org and https://seer.cancer.gov/statfacts, and the book *Cancer in Adolescents and Young Adults* – Chapter 13. Secondly, according to PRISMA Criteria Statement 2009 (Fig. 1; Moher *et al*., 2009) we systematically retrieved articles describing EO‐CRC prognosis compared with older counterparts, and ther prevalence of *RAS* and *BRAF* mutations through PubMed database using [young(Title) OR younger(Title) OR early‐onset(Title) OR adolescent(Title) OR association of age(Title)] AND [colon(Title) OR rectal(Title) OR colorectal(Title)] AND [cancer(Title)] as search terms. Articles published in languages other than English, published earlier than year 2000, articles with a follow up shorter than 2 years, articles including patients older than 50 years of age in the younger group, and articles not comparing young with old patients in the same cohort were not included in the systematic review. This search was implemented by further manual revision of the bibliography of the included articles.

### 2.1 Definition of age groups

An unequivocal definition of ‘EO‐CRC’, or ‘young adult CRC’, is currently needed, as no clear and widely accepted consensus is available in literature or guidelines. According to a non‐pediatric oncology definition, the definition generally comprises all CRCs diagnosed before the screening age, i.e. < 50 years of age. Most screening programs start from this age chosen based on cost‐effective analyses of healthcare system sustainability. In contrast, in Adolescent and Young Adult (AYA) Oncology, it comprises CRC patients diagnosed at 15–29 years of age (Bleyer *et al*., 2017). Nevertheless, in the context of some AYA clinical trials, the age range has been extended to 50 years by the Children's Oncology Group (Bleyer *et al*., 2017). The US National Cancer Institute (NCI) Progress Review Group of AYA Oncology in 2006 proposed the age range of 15–39 years (Bleyer *et al*., 2017; Coccia *et al*., 2018). The definition of ‘very EO‐ CRC’ has also no clear consensus in the literature, with extreme variability among different publications. Therefore, definition of age groups among CRC patients is currently based on non‐specific epidemiologic screening or clinical trial accrual criteria. Current age‐group subdivision is indeed a limitation for interpreting results obtained from published molecular and clinical studies among EO‐CRCs. In this review, we included all publications dealing with EO‐CRC diagnosed before 50 years of age.

A reasonable solution to improve future retrospective analyses of previously published studies in such a population of EO‐CRCs might be to consider age a continuous variable rather than using arbitrary predefined age cut‐offs (Lieu *et al*., 2014). The open question remains as to how to define age cut‐off for clinical trials.

### 2.2 Epidemiology

According to the Surveillance, Epidemiology and End Results Program (SEER) database in the USA, around 5% of all CRC are diagnosed in patients < 45 years old (Colorectal Cancer ‐ Cancer Stat Facts, 2018). Rectal cancer is diagnosed in up to 18% of cases < 50 years old in men and women alike (Ahnen *et al*., 2014). EO‐CRC is more commonly diagnosed among minorities and uninsured populations (You *et al*., 2012).

**Figure 2:**
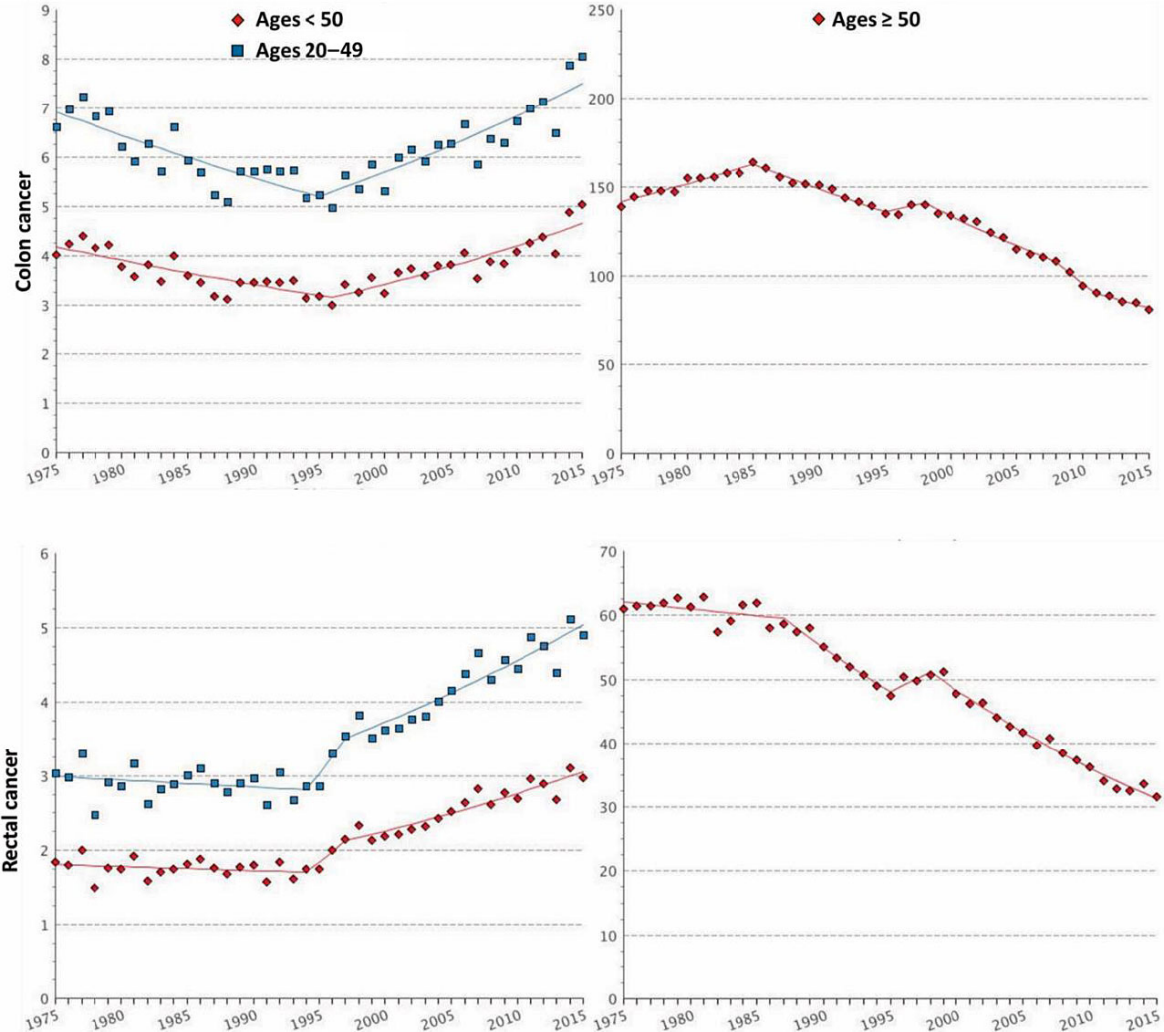
Graphs report age‐adjusted SEER (Surveillance, Epidemiology and End Results) incidence rates of colon (upper panels) and rectal (lower panels) cancer from 1975 to 2015 among individuals younger (left panels) and older (right panels) than 50 years. On the *Y*‐axis is reported incidence rate per 100 000 and on the *X*‐axis is reported the year of diagnosis. Data were plotted by accessing SEER website at the weblink https://seer.cancer.gov/faststats/selections.php?series=cancer

**Figure 3:**
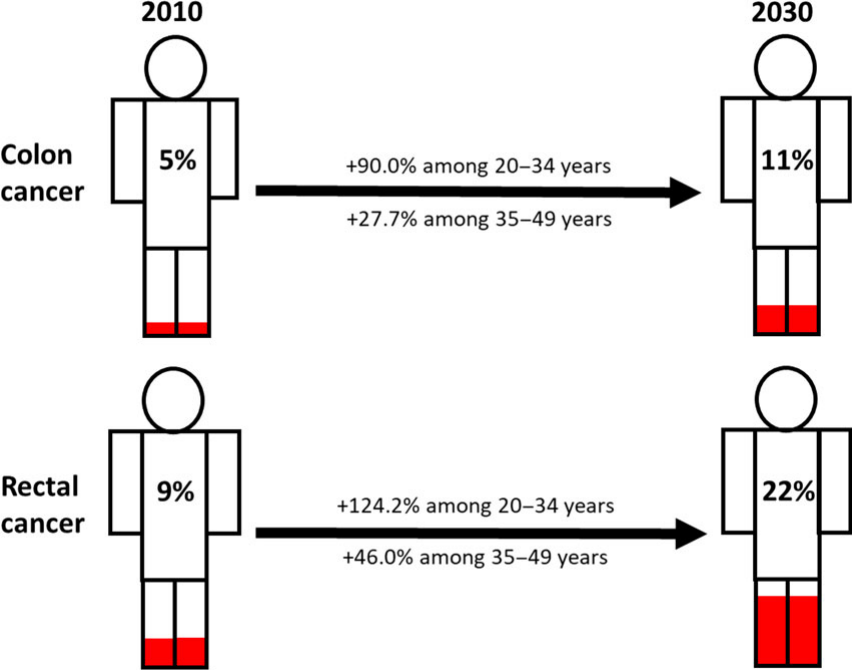
Incidence rates increase in colon and rectal cancer in patients younger than 50 years from 2010 to 2030 according to Bailey *et al*. (2015). Data and projections are confirmed for men and women in the USA). Figures colored in red represent percentages of CRC diagnosed under the age of 50.

**Figure 4:**
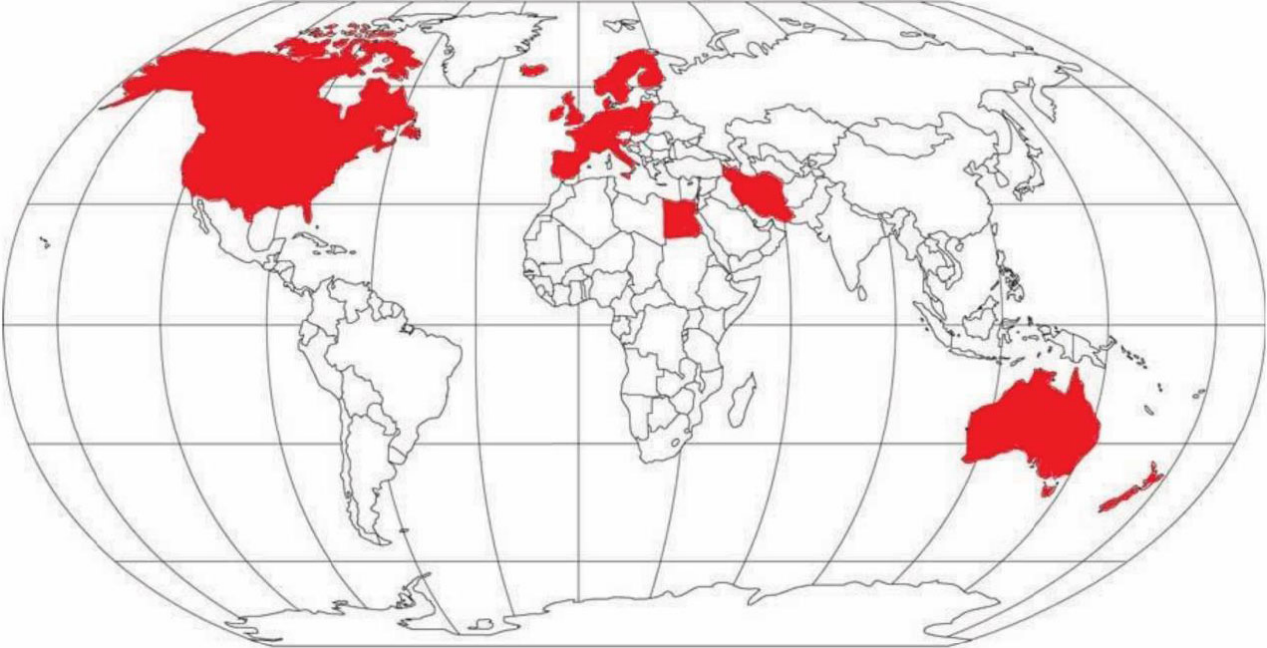
Map of the world and countries (shown in red) in which increased incidence of CRC among patients younger than 50 years old has been documented as described in the text (Abou‐Zeid *et al*., 2017; Ahnen *et al*., 2014; Bailey *et al*., 2015; Brenner *et al*., 2017; Exarchakou *et al*., 2018; Gandhi *et al*., 2017; Hessami Arani and Kerachian, 2017; Malekzadeh *et al*., 2009; A.‐G. Russo, A. Andreano, A. Sartore‐Bianchi, G. Mauri, A. Decarli & S. Siena, personal communication; Troeung *et al*., 2017; Vuik *et al*., 2018).

At the beginning of the 21st century, several studies showed evidence that CRC incidence was changing across different age groups. Indeed, in the USA an increase in the number of CRC diagnoses in the population younger than 50 years has been described and, in particular, this increase was observed in patients aged 20–35 years (Bailey *et al*., 2015; Bhandari *et al*., 2017; Edwards *et al*., 2014; Meyer *et al*., 2010; O'Connell *et al*., 2003). Epidemiological evidence indicates that since 1994, CRC incidence in those < 55 years is increasing by 2% per year (Figs 2 and 3; Fast Stats, 2018; Siegel *et al*., 2018). Bailey *et al*., based on CRC incidence trends between 1975 and 2010 in the USA, predicted a nearly doubled incidence rate of CRC by 2030 in the population aged < 35 years, and the highest increase is expected for recto‐sigmoid cancers between the ages of 18 and 35 years (Bailey *et al*., 2015). By 2030 in the USA, 10% of all colon and 22% of all rectal cancers are expected to be diagnosed in patients < 50 years old, a provocative prediction if compared with 4% and 9% for colon and rectal cancer, respectively, 10 years ago (Fig. 3; Bailey *et al*., 2015). These data indicate that EO‐CRC is a current public health issue in the USA (Ahnen *et al*., 2014; Bailey *et al*., 2015) and elsewhere (Fig. 4; Abou‐Zeid *et al*., 2017; Brenner *et al*., 2017; Exarchakou *et al*., 2018; Gandhi *et al*., 2017; Hessami Arani and Kerachian, 2017; Malekzadeh *et al*., 2009; A.‐G. Russo, A. Andreano, A. Sartore‐Bianchi, G. Mauri, A. Decarli & S. Siena, personal communication; Troeung *et al*., 2017; Vuik *et al*., 2018). Following this trend, in the USA it has been proposed that CRC screening should start at 45 years rather than 50 years, but further data are needed to explore this option in real life (Peterse *et al*., 2018).

Similarly, recent data from Europe indicate an annual 1.5% increase in rectal cancer incidence between 1990 and 2008, and an annual 7.4% increase in colon cancer incidence between 2008 and 2016 (Vuik *et al*., 2018). In the Western world, it is reasonable to assume that access to screening procedures, i.e. FOBT and recto‐sigmoidoscopy or colonoscopy in the population > 50 years, can explain only partially the decreasing trend in CRC incidence (Edwards *et al*., 2014; Welch and Robertson, 2016). Indeed, the trend towards CRC incidence increase among young individuals has been reported not only in the USA and Europe but also in Iran and Egypt (Abou‐Zeid *et al*., 2017; Hessami Arani and Kerachian, 2017; Malekzadeh *et al*., 2009).

Finally, based on current data and projections for the next years it is reasonable to predict an increase of CRC cases diagnosed worldwide in individuals < 50 years for reasons that need to be investigated appropriately.

### 2.3 Clinical presentation

Excluding patients adhering to specific screening programs, CRC diagnosis in young individuals is usually carried out when symptoms appear (Hill *et al*., 2007; Karnak *et al*., 1999; Riaz *et al*., 2017). Painless bleeding could anticipate other CRC symptoms by 2–3 years (Riaz *et al*., 2017). However, CRC diagnosis in young adults is carried out on average 6 months later than symptom onset due to low level of suspiciousness by probands and clinicians, sense of invincibility in young adults, and lack of medical insurance (Bleyer *et al*., 2008; Hill *et al*., 2007; O'Connell *et al*., 2004b).

In 61% of patients < 50 years (Kneuertz *et al*., 2015) and up to 76% of patients < 30 years (Indini *et al*., 2017; Kam *et al*., 2004; Khan *et al*., 2016), CRC is diagnosed as stage III or IV, strikingly different from older CRC patients (46–50% diagnosed as stage III or IV; Ferrari *et al*., 2008; Khan *et al*., 2016; Kneuertz *et al*., 2015). EO‐CRCs are more frequently poorly differentiated G3 tumors, left‐sided and rectal (Chang *et al*., 2012; Indini *et al*., 2017; Kam *et al*., 2004; Khan *et al*., 2016; Kneuertz *et al*., 2015; Sultan *et al*., 2010; Wang *et al*., 2015a). Signet ring cell CRC, accounting for < 1% of all CRC, among younger patients account for 3–13% of cases, especially in those younger than 30 years (Chang *et al*., 2012; Khan *et al*., 2016; Kneuertz *et al*., 2015; Wang *et al*., 2015a; Yantiss *et al*., 2009). Young patients usually have a Charlson comorbidity index of 0 and no other medical issues (Kneuertz *et al*., 2015).

### 2.4 Risk factors

Non‐Mediterranean Western diet, obesity, little physical activity, high consumption of red and processed meat, and low fiber intake are the most relevant risk factors for developing CRC (Castelló *et al*., 2018; Edwards *et al*., 2014; Hessami Arani and Kerachian, 2017; Slattery *et al*., 1998, 2003; Thune and Lund, 1996). There is a recently increased incidence of obesity in the USA, especially among young patients, and, being a well‐known risk factor for CRC, this may have played a role in reducing the age of CRC onset (Aleksandrova *et al*., 2013; Bailey *et al*., 2015; Bassett *et al*., 2010; Hedley *et al*., 2004). The net contribution of these factors cannot be assessed (Bailey *et al*., 2015; Kim *et al*., 2018).

### 2.5 There are CRC risk factors well‐known to play a more important role among young individuals:

- Inflammatory bowel diseases increase of by two‐ to threefold the risk of CRC compared with general population, especially when diagnosed in early age (Triantafillidis *et al*., 2009).
- Known hereditary cancer‐predisposing syndromes or familial CRC is higher among EO‐CRCs (see next paragraph; Hampel *et al*., 2005; Liang *et al*., 2003; Losi *et al*., 2005; Sinicrope, 2018).
- Low adherence to specific screening programs in individuals with known cancer syndromes or familial CRC is also a major point in countries with a private health system or among populations with a low socioeconomic level (Hampel *et al*., 2005; Shin *et al*., 2017; U.S. Preventive Services Task Force, 2002; Vale Rodrigues *et al*., 2018).
- Prior abdominal irradiation (i.e. radiotherapy for curable pediatric malignancies): colonoscopy is recommended starting from 35 years of age or a decade following > 30 Gy radiation treatment to the pelvis (Hill *et al*., 2007).

### 2.6 Molecular etiology, hereditary and familial syndromes

In the general population of CRC there are three main pathways of carcinogenesis involved in the onset and development of CRC: chromosomal instability (CIN), microsatellite instability (MSI), and CpG island methylator phenotype (CIMP).

Among EO‐CRCs, as well as in the general population, CIN is the most common cause of CRC (Goel *et al*., 2010; Markowitz and Bertagnolli, 2009). In a variable percentage of cases depending on disease stage, CRC is caused by MSI (Lanza *et al*., 2006; Tran *et al*., 2011; Zaanan *et al*., 2018). There are two possible mechanisms leading to MSI‐CRC (Sinicrope, 2018):

- hereditary germline mutations occurring in mismatch repair (MMR) genes;
- tumor somatic hypermethylation of *MLH1*.

In all MSI‐CRC patients, referral for genetic counseling is recommended to screen Lynch syndrome (LS; Palomaki *et al*., 2009). LS predisposes to EO‐CRC and the percentage of MSI tumors has been observed to rise to up to 27%, especially in patients < 30 years (Khan *et al*., 2016). In contrast to the overall CRC population, among patients younger than 30 years the location of the primary tumor and histology of MSI‐CRCs do not differ from MSS‐CRC (Khan *et al*., 2016).

These mechanisms of carcinogenesis are not mutually exclusive and may coexist (Armaghany *et al*., 2012; Snover, 2011). A small proportion of MSI‐CRCs can also present CIN, whereas around 50% of MSS‐CRC are CIN‐negative (Banerjea *et al*., 2009; Sinicrope *et al*., 2006; Tang *et al*., 2004). The latter subgroup has been defined as Microsatellite and Chromosome Stable (MACS). These tumors are more frequently rectal or left‐sided, characterized by a poor prognosis and poor recognition by the immune system (Banerjea *et al*., 2009; Sinicrope *et al*., 2006; Tang *et al*., 2004). MACS‐CRCs have been described more frequently among young patients with a family history of CRC, but data in the literature are conflicting (Chan *et al*., 2001; Rex *et al*., 2012). MACS‐CRC has been associated with LINE‐1‐hypomethylation and CIMP‐low. Among MACS‐CRC, *BRAF* mutation or absent MLH1 expression are rare (Antelo *et al*., 2012; Cai *et al*., 2008; Silver *et al*., 2012). In addition, recent results from an extended somatic molecular investigation in a cohort of left‐sided and rectal EO‐CRC showed higher mutation rates of *NF1*,* POLE*,* SMAD4* and *BRCA2* (Puccini *et al*., 2018). Furthermore, mutations in left‐sided EO‐CRC genes involved in histone modification, such as *KDM5C*,* KMT2A*,* KMT2C*,* KMT2D* and *SET2D*, were reported more frequently (Puccini *et al*., 2018).

A prominent cause of EO‐CRC is the presence of a germline oncogene mutation, giving rise to a hereditary cancer syndrome. Prevalence of hereditary CRC syndromes among EO‐CRCs is influenced by the different age groups analyzed in different studies (Mork *et al*., 2015; Pearlman *et al*., 2017; Stoffel *et al*., 2018), with a higher prevalence among patients < 35 years old (Mork *et al*., 2015).

Four potential genetic scenarios should be considered among EO‐CRCs: known hereditary cancer syndromes, *de novo* germline hereditary cancer mutations, familial colorectal cancer and non‐hereditary and non‐familial CRC.

#### 2.6.1 Hereditary cancer syndromes

**Figure 5:**
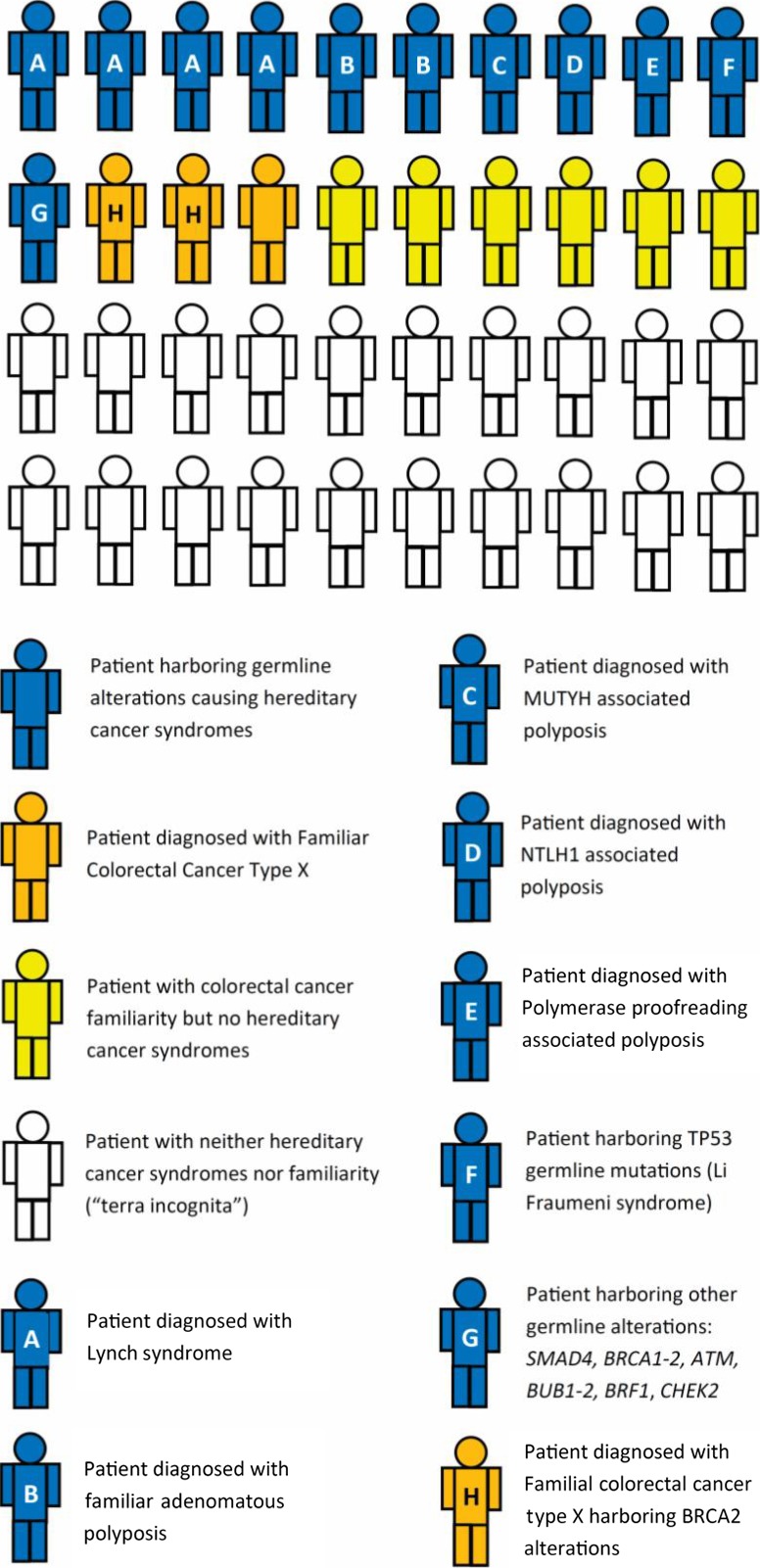
Prevalence of hereditary and familial syndromes, and neither hereditary nor familial syndromes (‘terra incognita’) among EO‐CRC in young individuals. Figures are derived from studies in the text (Bellido *et al*., 2018; Chang *et al*., 2012; Fong *et al*., 2009; Garre *et al*., 2015; Hahn *et al*., 2016; Jasperson *et al*., 2010; Ledermann *et al*., 2012; Lubbe *et al*., 2009; Mateo *et al*., 2015; Mork *et al*., 2015; Mur *et al*., 2018; Nejadtaghi *et al*., 2017; Palles *et al*., 2013; Pearlman *et al*., 2017; Robson *et al*., 2017; Sinicrope, 2018; Stoffel *et al*., 2018; Umar *et al*., 2004; Vasen *et al*., 1991, 1999; Weren *et al*., 2015, 2018; Yoshida *et al*., 2017; Yurgelun *et al*., 2015).

Around 22% (10–33% according to different studies) of patients diagnosed with EO‐CRC are affected by hereditary cancer syndromes (Fig. 5). This proportion is significantly higher when compared with the 2–5% of hereditary cancer syndromes among the general CRC population (Chang *et al*., 2012; Jasperson *et al*., 2010; Mork *et al*., 2015). The most frequent hereditary cancer syndrome is hereditary non‐polyposis colorectal cancer, also known as LS (Mork *et al*., 2015; Pearlman *et al*., 2017; Sinicrope, 2018; Stoffel *et al*., 2018). LS patients have a lifetime risk of developing CRC of 70%, and in 40% of cases the onset of CRC is before age 40 (Lynch *et al*., 2008). LS is the most frequent CRC hereditary syndrome among patients younger than 50 years old and accounts for around one‐third of EO‐CRC in patients younger than 35 years old (Mork *et al*., 2015; Pearlman *et al*., 2017; Stoffel *et al*., 2018). Germline mutations in *MLH1* and *MSH2* genes are the most frequent, but *MSH6* and *PMS2* gene can present pathogenic mutations as well (Evans *et al*., 2007; Lynch *et al*., 2015). In addition, mutations occurring in *EPCAM* gene can also silence the *MSH2* promoter region, leading to LS (Kempers *et al*., 2011; Ligtenberg *et al*., 2009, p. 2). In some cases of LS, homozygous or compound heterozygous (bi‐allelic) constitutive MMR gene mutations have been identified (Durno *et al*., 2005, 2015; Wimmer and Etzler, 2008). These lead to a very low age of CRC onset, usually earlier than 16 years old (Durno *et al*., 2005, Wimmer and Etzler, 2008). In contrast, a relevant proportion of young patients with MSI‐H tumors, usually MLH1 hypermethylated, do not harbor germline alterations in MMR genes (Pearlman *et al*., 2017; Zbuk *et al*., 2009).

CRC may develop earlier in patients affected by adenomatous polyposis syndromes: familial adenomatous polyposis (FAP), polymerase proofreading‐associated polyposis (PAPP), MutYH‐associated polyposis (MAP) and NTHL1‐associated polyposis (NAP). FAP is the second most frequent hereditary cancer syndrome (Mork *et al*., 2015). Lifetime risk of developing CRC in FAP patients is 100% with a median age of CRC onset of 39 years old (Zbuk *et al*., 2009). Different *APC* mutations have been associated with different phenotypes, with mutations occurring in codon 1309 being associated with severe polyposis in the first decade of life (Caspari *et al*., 1994). FAP screening is recommended from the age of 10 years old and the treatment of choice is total proctocolectomy (Warrier and Kalady, 2012; Winawer *et al*., 2003). PAPP is an autosomal dominant genetic disease leading to increased risk of cancer and by germline mutations in *POLE* and *POLD1* genes (Palles *et al*., 2013). PAPP confers a highly penetrant predisposition to develop polyposis (usually < 100) and EO‐CRCs (Palles *et al*., 2013; Valle *et al*., 2014). In addition, mutations occurring in *POLE* and *POLD1* can occur *de novo* and in patients without polyposis (Valle *et al*., 2014). Thus, alterations in these genes could be found in all familial CRCs and are not restricted to polyposis cases (Valle *et al*., 2014). Differently, MAP and NAP are two autosomal recessive genetic diseases caused by germline mutation in *MutYH* and *NTHL1* DNA glycosylase gene of the base excision repair (BER) pathway (Weren *et al*., 2015, 2018). These syndromes are characterized by the onset of 10–50 polyps around the age of 40 that cause an increased risk of CRC (Weren *et al*., 2015, 2018). MAP confers a 28‐fold higher lifetime risk of developing CRC at usually < 60 years old with a reported penetrance of 40–100% (Lubbe *et al*., 2009; Weren *et al*., 2018).

Other cancer syndromes, such as Li‐Fraumeni syndrome, characterized by germline *TP53* mutations, have been related to EO‐CRC; they account for less than 1% of cases (Yoshida *et al*., 2017; Yurgelun *et al*., 2015; Fig. 5).

In patients with known hereditary syndromes, adherence to screening programs should be strongly encouraged, as in LS it has been demonstrated to reduce the risk of death by 65% (Järvinen *et al*., 2000). However, screening program adherence is low in youths, including in Western countries, and has to be enhanced to reduce late diagnosis and cancer deaths (Kim *et al*., 2017; Shaikh *et al*., 2018). Furthermore, a preventive aspirin regimen has been reported to significantly reduce CRC incidence among LS patients (Burn *et al*., 2011).

#### 2.6.2 Other germline alterations associated with EO‐CRC

Many recent studies have tried to broaden the spectrum of germline alterations predisposing to EO‐CRC to expand the population admitted to screening programs. Several genes have been investigated, e.g. *SMAD4, BRCA1‐2, ATM, BUB1‐2, BRF1* and *CHEK2* (Bellido *et al*., 2018, p. 1; Hahn *et al*., 2016; Mur *et al*., 2018; Pearlman *et al*., 2017; Stoffel *et al*., 2018; Yurgelun *et al*., 2015); however, at around 2–10% they account for only a small proportion of EO‐CRC overall (Pearlman *et al*., 2017; Stoffel *et al*., 2018). Although rare, these genetic alterations should not be neglected, especially for those that can potentially be treated with drugs, such as *BRCA2* alterations (Fong *et al*., 2009; Ledermann *et al*., 2012; Mateo *et al*., 2015; Robson *et al*., 2017). As pointed out by different studies, a large percentage of EO‐CRC patients with no CRC familiarity may be affected by hereditary cancer syndromes or at least harbor gene alterations potentially predisposing to CRC (Mork *et al*., 2015; Stoffel *et al*., 2018). Only half (43/85) of EO‐CRC patients presenting germline cancer‐predisposing alterations reported CRC in a first‐degree relative, therefore being the first to be diagnosed with a cancer‐predisposing syndrome (Stoffel *et al*., 2018). This evidence has led some authors to recommend genetic counseling for all patients diagnosed with extreme EO‐CRC (< 35 years old; Mork *et al*., 2015).

#### 2.6.3 Familial EO‐CRC

The opposite situation is represented by patients characterized by a relevant familial CRC not affected by hereditary CRC syndromes. In particular, this is the case for patients fulfilling Amsterdam Criteria I or II but not affected by LS (Garre *et al*., 2015; Vasen *et al*., 1991, 1999). Today, the Amsterdam criteria have largely been replaced by the Bethesda criteria, which are more sensitive but less specific in identifying *MSH2* and *MLH1* germline mutations carriers, thus broadening the spectrum of familial CRC patients (Piñol *et al*., 2005; Umar *et al*., 2004). Amsterdam or Bethesda criteria‐positive patients without a diagnosis of LS are diagnosed with Familiar Colorectal Cancer Syndrome Type X (Nejadtaghi *et al*., 2017; Umar *et al*., 2004; Vasen *et al*., 1991, 1999). This peculiar population may be an ideal group in whom to extend molecular screening in order to find out potentially actionable gene alterations hopefully to confer survival benefit, if receiving appropriately targeted agents (Garre *et al*., 2015; Nejadtaghi *et al*., 2017). In this regard, Garre and coworkers documented that, in a population of EO‐CRC patients fulfilling Amsterdam criteria II, the prevalence of *BRCA2* germline mutations, including point and frame mutations, was 60% (29/48; Garre *et al*., 2015), leading to the speculation that these patients might derive benefit from platinum‐ or PARP‐inhibitor‐based regimens in the metastatic setting (Garre *et al*., 2015; Mylavarapu *et al*., 2018).

In addition, CRC incidence in this subgroup may increase in the next years due to the activity of known carcinogenic agents in patients harboring a hypothetical genetic predisposition to develop CRC. At this regard, in a Japanese cross‐sectional study it has been recently reported that alcohol consumption might be associated with earlier CRC onset in patients affected by LS (Miguchi *et al*., 2018).

#### 2.6.4 Non‐hereditary and non‐familial EO‐CRC

It is very surprising to note that although hereditary and familial cancer syndromes are more frequent among young individuals, half of EO‐CRC patients have neither (Mork *et al*., 2015; Stoffel *et al*., 2018). This implies that, since these patients are not included in screening programs, they are often diagnosed in later stages of the disease. Among this subset of patients, without known hereditary cancer syndromes, different alterations in *TNFR1, EIF4E, LTBP4, CYR61, UCHL1, FOS* and *FOS B* genes have been demonstrated between early‐ and late‐onset CRC. However, definite conclusions are still far from being reached, since study populations were limited to cohorts of 10–39 patients and the gene panels tested were heterogeneous (Berg *et al*., 2010; Hong *et al*., 2007; Kirzin *et al*., 2014).

In addition to somatic tumor mutations, tumor immune infiltration is currently under investigation in EO‐CRC. Recently, in EO‐CRC a different expression of immune activation genes (i.e. *CLC* and *IFNAR1*) has been reported when compared with the older counterparts (Ågesen *et al*., 2011). Overall, it is conceivable that among EO‐CRCs, more patients could potentially benefit from immunotherapy in the metastatic setting because of a higher prevalence of MMR, POLE and POLD1 aberrations, leading to a higher tumor mutational burden (Pai *et al*., 2017). However, data on peculiar features of interactions between the immune system and tumors in EO‐CRC are still to be addressed.

### 2.7 What could be responsible for sporadic EO‐CRC?

Environmental reasons for EO‐CRC are still unknown. In the last decades, dramatic environmental and behavioral changes may have contributed to lowering the age of CRC onset. Changes in microbiome induced by cesarean delivery or appendicectomy might play a role. Microbiome may also be altered by modern dietary regimens including colorants and preservatives. These substances may also play a role of direct carcinogenesis on intestine cells. The extensive use of antibiotics in agriculture and medicine, although immediately beneficial, could reasonably alter the gut microbiome. Reduced breast‐feeding may alter the development of the immune system and its capacity for cancer surveillance. However, we still do not know how these agents may alter cells pathways causing CRC. Large epidemiological and preclinical studies are warranted.

### 2.8 Prognosis, treatments, and outcome

We systematically retrieved 37 articles describing the prognosis of EO‐CRC compared with older patients (Abdelsattar *et al*., 2016; Blanke *et al*., 2011; Boyce *et al*., 2016; Chandrasinghe *et al*., 2017; Chou *et al*., 2011, 2017; Damodaran and Seshadri, 2016; Fu *et al*., 2014; Fu *et al*., 2013; Haleshappa *et al*., 2017; Hawk *et al*., 2015; Hubbard *et al*., 2012; Josifovski *et al*., 2004; Khan *et al*., 2016; Kim *et al*., 2016; Kneuertz *et al*., 2015; Kolarich *et al*., 2018; Li *et al*., 2014; Lieu *et al*., 2014; Manjelievskaia *et al*., 2017; McMillan and McArdle, 2009; Murata *et al*., 2016; O'Connell *et al*., 2004a; Orsini *et al*., 2015; Pokharkar *et al*., 2017; Quah *et al*., 2007; Rho *et al*., 2017; Rodriguez *et al*., 2018; Shen *et al*., 2018; Shida *et al*., 2018; Sultan *et al*., 2010; Vatandoust *et al*., 2016; Wang *et al*., 2015a,2015b; Yang *et al*., 2012; You *et al*., 2012; Zhao *et al*., 2017). EO‐CRC survival data are conflicting. Some studies indicate a poorer prognosis (Bleyer *et al*., 2008; Khan *et al*., 2016; Lieu *et al*., 2014; McMillan and McArdle, 2009; O'Connell *et al*., 2004b; Shida *et al*., 2018; Sultan *et al*., 2010), whereas others support a comparable or even better prognosis in comparison with older patients (Blanke *et al*., 2011; Hubbard *et al*., 2012; Kneuertz *et al*., 2015; Kolarich *et al*., 2018; McMillan and McArdle, 2009; O'Connell *et al*., 2004a; Quah *et al*., 2007; Rodriguez *et al*., 2018; Vatandoust *et al*., 2016; Wang *et al*., 2015a; You *et al*., 2011; Table 1). From 1973 to 2005, adult CRC survival outcome improved, whereas child and adolescent CRC survival have not (Sultan *et al*., 2010). A worse survival was observed in studies comparing survival between patients younger than 30 years old with those > 50 years (Khan *et al*., 2016; Lieu *et al*., 2014; Sultan *et al*., 2010). In particular, it was reported that the younger the patient, the worse the prognosis (Lieu *et al*., 2014). By contrast, publications comparing patients younger than 50 years of age with those > 50 years showed a better prognosis among the younger patients (Kneuertz *et al*., 2015; Kolarich *et al*., 2018; Vatandoust *et al*., 2016; Wang *et al*., 2015a). This suggests that in patients with very early‐onset CRC (< 35 years old) a different biological background likely underlies an earlier and faster CRC progression (Ferrari *et al*., 2008; Indini *et al*., 2017; Khan *et al*., 2016; Zhao *et al*., 2017).

**Table 1:** Prognosis of early‐onset colorectal cancer patients according to age groups from systematically reviewed studies written in English, published later than 2000 with a follow up longer than 2 years and considering 50 years of age as upper cut‐off of early‐onset, according to the PRISMA Criteria of 2009 (Moher *et al*., 2009). Color codes: Green, significantly better prognosis; Yellow, similar prognosis; Red, significantly worse prognosis. ARR, adjusted relative risk; CSS, cancer‐specific survival; DSS, disease‐specific Survival; HR, hazard ratio; LC, Linköping cancer; MHS, Military Health System; mOS, median overall survival; NSS, not statistically significant; NSW, New South Wales; NCDB, National Cancer Data Base; OS, overall survival; PD, progressive disease; RER, relative excess risk; SAMCRC, South Australian Metastatic Colorectal Cancer; SEER, Surveillance, Epidemiology and End Results program

Authors (references)	Data source	Stage of disease	Age groups (range in years of age)	Prognosis
Kneuertz *et al*. (2015)	NCDB	Stage II	18–49	5‐year ARR 0.72 (0.58–0.88)
			65–75	
		Stage II	18–49	5‐year ARR 0.90 (0.69–1.17)
			65–75	
		Stage III	18–49	5‐year ARR 0.89 (0.81–0.97)
			65–75	
		Stage IV	18–49	5‐year ARR 0.84 (0.79–0.90)
			65–75	
McMillan and McArdle (2009)	Hospital records	All stages	< 45	10‐year CSS *P *=* *0.275
			> 45	
Khan *et al*. (2016)	Hospital records	All stages	< 30	5‐year DSS *P *<* *0.0001
			> 50	
Shen *et al*. (2018)	Hospital records	Stage I‐III	< 35	5‐year OS *P *=* *0.0013^a^
			> 35	
O'Connell *et al*. (2004a)	SEER (1991–1999)	Stage I	< 40	CSS *P *=* *NS
			60–80	
		Stage II	< 40	CSS *P *=* *0.01
			60–80	
		Stage III	< 40	CSS *P *=* *NS
			60–80	
		Stage IV	< 40	CSS *P *<* *0.0001
			60–80	
Sultan *et al*. (2010)	SEER (1973–2005)	All stages	< 20	OS *P *<* *0.001
			> 20	
You *et al*. (2012)	Hospital records	Stage I	< 50	5‐year CSS *P *=* *NS
			> 65	
		Stage II	< 50	5‐year CSS *P *=* *0.012
			> 65	
		Stage III	< 50	5‐year CSS *P *=* *NS
			> 65	
		Stage IV	< 50	5‐year CSS *P *=* *NS
			> 65	
Wang *et al*. (2015a)	SEER (1973–2011)/LC (1972–2009)	Stage I	< 50	CSS *P *<* *0.001
			> 50	
		Stage II	< 50	CSS *P *<* *0.001
			> 50	
		Stage III	< 50	CSS *P *<* *0.001
			> 50	
		Stage IV	< 50	CSS *P *<* *0.001
			> 50	
Wang *et al*. (2015b)	SEER (1988–2011)	Stage I	< 50	CSS *P *<* *0.001
			> 50	
		Stage II	< 50	CSS *P *<* *0.001
			> 50	
		Stage III	< 50	CSS *P *<* *0.001
			> 50	
		Stage IV	< 50	CSS *P *<* *0.001
			> 50	
Quah *et al*. (2007)	Hospital records	Stage I‐III	< 40	5‐year DSS p.43
			> 40	
Murata *et al*. (2016)	Hospital records	Stage I‐III	< 40	5‐year OS *P *=* *0.93
			≥ 40	
Vatandoust *et al*. (2016)	SAMCRC database	Stage IV	< 40	mOS HR 0.81 (0.56–1.16)
			> 40	
Kolarich *et al*. (2018)	NCDB	Stage I	< 50	5‐year OS *P *<* *0.001
			> 50	
		Stage II	< 50	5‐year OS *P *<* *0.001
			> 50	
		Stage III	< 50	5‐year OS *P *<* *0.001
			> 50	
Rodriguez *et al*. (2018)	Ontario registry	Stage I‐III	< 40	5‐year OS *P *<* *0.001
			> 60	
Orsini *et al*. (2015)	Netherlands cancer registry	Stage I‐III	≤ 40	RER of death 0.82 (0.71–0.94)
			> 40	
			≤ 40	RER of death 1.04 (0.91–1.18)
			> 40	
Damodaran and Seshadri (2016)	Hospital records	Stage II‐III	≤ 40	5‐year CSS *P *=* *NSS
			> 40	
Blanke *et al*. (2011)	Clinical trials	Stage IV	< 40	mOS *P *=* *0.61
			< 40	
			< 50	mOS *P *=* *0.48
			< 50	
Hubbard *et al*. (2012)	Clinical trials	Stage II	< 40	OS *P *<* *0.01
			> 40	
		Stage III	< 40	OS *P *<* *0.01
			> 40	
Shida *et al*. (2018)	Hospital records	Stage IV	< 40	5‐year OS *P *=* *0.042
			> 40	
Haleshappa *et al*. (2017)	Hospital records	All stages	< 40	mOS *P *=* *0.0029
			> 40	
Pokharkar *et al*. (2017)	Hospital records	All stages	< 45	3‐year OS *P *=* *0.302
			> 45	
Chou *et al*. (2017)	Taiwan cancer registry	All stages	< 40	10‐year CRC related mortality *P *<* *0.001
			41–70	
Chandrasinghe *et al*. (2017)	Hospital records	All stages	< 50	5‐year OS *P *=* *0.03
			> 70	
Rho *et al*. (2017)	Hospital records	All stages	18–44	Mortality risk HR 1.53 (0.91‐ 2.58)
			> 44	
Zhao *et al*. (2017)	Hospital records	Stage I‐III	≤ 35	5‐year OS *P *=* *0.010
			> 35	
Manjelievskaia *et al*. (2017)	US MHS database	Stage I	18–49	5‐year survival HR 0.29 (0.13–0.62)^b^
			50–64	
		Stage II	18–49	5‐year survival HR 0.59 (0.31–1.14)^b^
			50–64	
		Stage III	18–49	5‐year survival HR 0.01 (0.01–0.89)^b^
			50–64	
		Stage IV	18–49	5‐year survival HR 0.47 (0.22–0.98)^b^
			50–64	
		Stage I‐IV	18–49	5‐year survival HR: NSS c
			50–64	
Boyce *et al*. (2016)	NSW central cancer registry	All stages	< 50	5‐year CSS *P *<* *0.001
			> 50	
Kim *et al*. (2016)	Hospital records	Stage I	22–45	5‐year CSS *P *=* *0.188
			56–75	
			22–45	5‐year CSS *P *=* *0.771
			56–75	
			22–45	5‐year CSS *P *=* *0.087
			56–75	
			22–45	5‐year CSS *P *=* *0.142
			56–75	
Abdelsattar *et al*. (2016)	SEER (1998–2011)	Stage I‐II	< 50	5‐year CSS *P *<* *0.001
			> 50	
		Stage III	< 50	5‐year CSS *P *<* *0.001
			> 50	
		Stage IV	< 50	5‐year CSS *P *<* *0.001
			> 50	
Fu *et al*. (2014)	Hospital records	Stage I‐III	≤ 35	10‐year OS *P *=* *NSS
			> 35	
		Stage IV	≤ 35	10‐year OS *P *=* *0.046
			> 35	
Li *et al*. (2014)	SEER (1988–2003)	Stage I‐III	≤ 40	5‐year CSS *P *<* *0.001
			> 40	
Hawk *et al*. (2015)	SEER (1973–2008)	Stage IV	< 50	OS HR 0.725 (0.703–0.749)
			> 50	
Fu *et al*. (2013)	Hospital records	Stage I‐II	≤ 30	10‐year OS *P *=* *0.899
			> 30	
		Stage III‐IV	≤ 30	10‐year OS *P *=* *0.024
			> 30	
Yang *et al*. (2012)	Hospital records	All stages	≤ 44	10‐year OS *P *=* *NSS
			> 44	
Chou *et al*. (2011)	Hospital records	All stages	≤ 40	5‐year CSS *P *<* *0.001
			≥ 80	
Josifovski *et al*. (2004)	Hospital records	All stages	< 40	5‐year OS *P *=* *0.053
			> 65	
Lieu *et al*. (2014)	Clinical trials	Stage IV	≈ 18^d^	+19% risk of death +22% risk of PD
			≈ 57–61^d^	

a Worse 5‐year OS was observed only in female and young colorectal cancer patients.

b Patients treated with surgery alone.

c Patients treated with surgery and postoperative chemotherapy.

d No age cut‐offs available: age used a continuous variable rather than using specified cut‐off points.

Clinical guidelines do not consider early age of onset of CRC as a criterion to drive treatment, either in the adjuvant or in the metastatic setting. To date, therapeutic options for early‐ and late‐onset CRC patients are the same according to the major oncology societies worldwide (Benson *et al*., 2017; Van Cutsem *et al*., 2014; Yoshino *et al*., 2018). However, many publications describe a more aggressive attitude of both clinicians and surgeons treating early‐onset stage III and IV CRC (Khan *et al*., 2016; Kneuertz *et al*., 2015). This aggressiveness has been demonstrated to not confer a significant survival benefit (Kneuertz *et al*., 2015). In particular, an adjuvant, more intensive schedule of chemotherapy or targeted agents failed to confer a survival benefit and, overall, a more aggressive surgical and adjuvant approach in young CRC patients should not be recommended, since it may lead to overtreatment (Alberts *et al*., 2012; Allegra *et al*., 2011; De Gramont *et al*., 2012; Kneuertz *et al*., 2015; Taieb *et al*., 2017). Combining neo‐adjuvant chemo‐radiotherapy with surgery failed to confer a survival advantage in rectal patients younger than 50 years of age when compared with surgery alone (Kolarich *et al*., 2018). Conversely, oxaliplatin added to standard chemoradiotherapy in patients affected by locally advanced rectal cancer seems to improve disease‐free survival and OS in those < 60 years of age (Hofheinz *et al*., 2018).

In the metastatic setting, two recent retrospective analyses have analyzed the outcome of EO‐CRC. A retrospective study including nine phase III trials stated that patients < 40 years old at diagnosis had a poorer progression‐free survival (PFS) but not OS or response rate (RR) in comparison with patients > 50 years (Blanke *et al*., 2011). More recently, Lieu and coworkers considered age as a continuous variable rather than a prespecified cut‐off and pooled results of 24 first line metastatic CRC trials including young patients treated with anti‐EGFR treatment according to CAIRO2, COIN, FIRE II and PRIME studies (Lieu *et al*., 2014). They reported that EO‐CRC patients diagnosed in the 1920s experienced a worse PFS and OS when compared with patients diagnosed in their middle ages. They stated that prognostic effect of age did not differ according to class of targeted therapy for either OS or PFS.

Finally, age‐grouped comparisons assessing the efficacy of triplet vs doublet drug combinations in young patients are still lacking. Even if Lieu *et al*. were the first to include patients treated with anti‐EGFR drugs, more molecularly focused analyses are warranted to clarify this complex picture. Whether age of onset in addition to sidedness is a factor involved in determining anti‐EGFR drug resistance is still unknown (Boeckx *et al*., 2017; Holch *et al*., 2017).

### 2.9 *RAS* and *BRAF* mutations among EO‐CRC

Similar to all‐age populations, anti‐EGFR drugs are a valid treatment option for *RAS* wild‐type EO‐CRC patients (Benson *et al*., 2017; Douillard *et al*., 2013; Van Cutsem *et al*., 2009, 2016; Yoshino *et al*., 2018). *RAS* mutations are identified in 40% of all CRC (Cancer Genome Atlas Network, 2012).

We systematically retrieved 11 articles describing *RAS* and *BRAF* prevalence among EO‐CRC; data are conflicting (Alsop *et al*., 2006; Chang *et al*., 2012; Goel *et al*., 2010; Khan *et al*., 2016; Kirzin *et al*., 2014; Magnani *et al*., 2015; Perea *et al*., 2017; Rho *et al*., 2017; Tsai *et al*., 2016; Watson *et al*., 2016; Yantiss *et al*., 2009; Table 2). Most studies revealed a lower prevalence of *KRAS* mutations among EO‐CRC (4–31%), but others reported a similar (35–39%) or higher (54%) prevalence to that in older patients (Alsop *et al*., 2006; Chang *et al*., 2012; Goel *et al*., 2010; Khan *et al*., 2016; Kirzin *et al*., 2014; Magnani *et al*., 2015; Perea *et al*., 2017; Rho *et al*., 2017; Watson *et al*., 2016; Yantiss *et al*., 2009; Table 2).

**Table 2:** Assessment of primary tumor side, *KRAS, NRAS,* and *BRAF* mutations among early‐onset colorectal cancer (EO‐CRC)

Author (references)	Age cut‐off	Primary tumor in left colon or rectum (%)	*KRAS* mutation prevalence (%)	*KRAS* codons analyzed	*NRAS* mutation prevalence (%)	*NRAS* codons analyzed	*BRAF V600E* mutation prevalence (%)
Chang et al. (2012)	40	44/55 (80)	2/45 (4)	12, 13, 61	N/A	N/A	0/45 (0)
Yantiss et al. (2009)	40	22/24 (91)	6/24 (25)	12, 13	N/A	N/A	2/24 (8)
Goel et al. (2010)	50	54/75 (72)	18/66 (27)	12, 13	N/A	N/A	0/66 (0)
Alsop et al. (2006)	45	4/6 (67) *	6/101 (6)	12, 13, 61	N/A	N/A	N/A
Watson et al. (2016)	40	42/68 (62)	37/68 (54)	12, 13, 61	1/14 (1)	12, 13, 61	0/17 (0)
Khan et al. (2016)	30	62/94 (66)	26 (28)	12, 13	N/A	N/A	8 (9)
Tsai et al. (2016)	30	N/A	N/A	N/A	N/A	N/A	11/66 (19)
Kirzin et al. (2014)	45	36/48 (75)	17/48 (35)	12, 13	N/A	N/A	0/48
Rho et al. (2017)	44	152/224 (68)	24/77 (31)	N/A	N/A	N/A	N/A
Magnani et al. (2015)	30	26/33 (79)	10/33 (30)	12,13, 61	N/A	N/A	0/33 (0)
Perea et al. (2017)	45	N/A **	27/69 (39)	12, 13, 61	3/69 (0.5)	N/A	N/A

*Sidedness was reported only for KRAS mutant early‐onset colorectal cancer.

**In the article a general left sided predominance is reported without figures of prevalence percentage.

N/A = not available.

[Correction added after online publication on 21 January 2019: Table 2 corrected]

*NRAS* prevalence was reported at around 1% in a small population (*n *=* *69) of EO‐CRC (Perea *et al*., 2017). The prevalence of *BRAF* mutations is reported to be similar among EO‐CRC and the older onset patients, ranging from 0 to 19% (Chang *et al*., 2012; Goel *et al*., 2010; Khan *et al*., 2016; Kirzin *et al*., 2014; Magnani *et al*., 2015; Tsai *et al*., 2016; Watson *et al*., 2016; Yantiss *et al*., 2009).

In conclusion, as summarized in Table 2, there are no unequivocal data regarding the prevalence of *RAS/RAF* mutations in EO‐CRC.

### 2.10 Considerations for future perspectives

Most of the studies on EO‐CRC are retrospective and include a small number of patients of different ranges of ages. Despite the wide heterogeneity, the reviewed studies can be considered to generate hypotheses, even though no definitive conclusions or recommendations can be derived. Several studies demonstrate an increase incidence of EO‐CRC in different areas of the world, but the reasons behind this epidemiologic phenomenon are still to be unveiled. To counteract the greatest loss of life‐years in this younger than average CRC population, we need to achieve the best possible clinical results with the current treatments and to develop new therapies. Non‐hereditary CRC among the youngest (< 35 years old) represents the toughest challenge and this subset of EO‐CRC is expected to become even more prevalent in the next years. Available data suggest that survival is worst in patients younger than 30 years old, whereas it is comparable or even better among patients between 40 and 50 when compared with those older than 50 years. Given the clinical and molecular peculiarities of EO‐CRC, especially in patients younger than 30 years of age, a different molecular carcinogenesis might be speculated in those sporadic cases.

A mandatory step to answering questions of recognizing EO‐CRC as a common subdivision of age groups is necessary for comparison of EO‐CRC studies. In interpreting retrospective studies, considering age as a continuous variable may be an interesting solution. In addition, clinical criteria could be useful to identify EO‐CRC patients more likely to benefit from targeted therapy, such as MSS *BRCA2* mutated patients fulfilling Amsterdam criteria, or immunological treatments, such as patients harboring POLE or POLD hypermutated tumors potentially benefitting from checkpoint inhibitors. The role of environmental risk factors and the microbiome remains to be clarified. Finally, but more compelling, there is still a scarcity of clinical trials focusing on EO‐CRC and these are warranted to improve care of these patients.

## Conflict of interest

AS‐B has acted as a consultant/advisory member for Amgen, Bayer, Lilly and Merck‐Serono. AB has acted as a consultant/advisory member for Horizon Discovery, Biocartis and Trovagene. SS is an advisory board member for Amgen, Bayer, BMS, CheckmAb, Celgene, Incyte, Merck, Novartis, Roche and Seattle Genetics.
